# Statistical methods versus machine learning techniques for donor-recipient matching in liver transplantation

**DOI:** 10.1371/journal.pone.0252068

**Published:** 2021-05-21

**Authors:** David Guijo-Rubio, Javier Briceño, Pedro Antonio Gutiérrez, Maria Dolores Ayllón, Rubén Ciria, César Hervás-Martínez

**Affiliations:** 1 Department of Computer Sciences and Numerical Analysis, University of Córdoba, Córdoba, Spain; 2 Unit of Hepatobiliary Surgery and Liver Transplantation, Hospital Universitario Reina Sofía, IMIBIC, Córdoba, Spain; University of Toledo, UNITED STATES

## Abstract

Donor-Recipient (D-R) matching is one of the main challenges to be fulfilled nowadays. Due to the increasing number of recipients and the small amount of donors in liver transplantation, the allocation method is crucial. In this paper, to establish a fair comparison, the United Network for Organ Sharing database was used with 4 different end-points (3 months, and 1, 2 and 5 years), with a total of 39, 189 D-R pairs and 28 donor and recipient variables. Modelling techniques were divided into two groups: 1) classical statistical methods, including Logistic Regression (LR) and Naïve Bayes (NB), and 2) standard machine learning techniques, including Multilayer Perceptron (MLP), Random Forest (RF), Gradient Boosting (GB) or Support Vector Machines (SVM), among others. The methods were compared with standard scores, MELD, SOFT and BAR. For the 5-years end-point, LR (AUC = 0.654) outperformed several machine learning techniques, such as MLP (AUC = 0.599), GB (AUC = 0.600), SVM (AUC = 0.624) or RF (AUC = 0.644), among others. Moreover, LR also outperformed standard scores. The same pattern was reproduced for the others 3 end-points. Complex machine learning methods were not able to improve the performance of liver allocation, probably due to the implicit limitations associated to the collection process of the database.

## Introduction

Donor-Recipient (D-R) matching is one of the most challenging topics in Liver Transplantation (LT). Considering the increasing number of candidates for LT and the scarce number of available donors, the rationale for assignment of a given donor to potential candidates on a waiting list is a matter of controversy. For this purpose, some scores have been designed, whose implementation in practice has its supporters and detractors. Model for End-Stage Liver Disease (MELD) [1], Survival Following Liver Transplantation score (SOFT) [2] or Balance of Risk (BAR) [3] are examples of the intention to match donors and recipients to obtain the best post-transplant result. However, this result is also a subject of discussion. For some of these scores, the main objective is to decrease the mortality in the waiting list without affecting the result of the transplant. This is the case of MELD, the most widespread prioritization system nowadays. On the contrary, in other scores the idea of obtaining the greatest survival benefit prevails, which means combining the lowest mortality in the list with the best possible result. Unfortunately, none of these systems has been able to combine the urgency of a given transplant candidate with the best survival benefit among possible candidates. A decrease in mortality in the waiting list leads, in many cases, to worse post-transplant survival results; and, vice versa, obtaining better results may affect the opportunity to obtain a transplant for the most critical receipt on the waiting list.

In essence, D-R matching is a classification problem, where some variables of the donor are combined with variables of the listed recipients, surgical aspects and logistics factors to, in short, obtain a survival prediction [4]: survival of the graft, survival of the recipient, or both. A common problem of the available scores is a remarkably basic statistical methodology that only considers isolated variables with single random grafts or patient survival end-points. The combination of several variables and end-points, in the setting of artificial intelligence-based decisions that avoid human-guided bias, may be the basis for D-R matching and grafts allocation in the future.

In this paper, we aim to analyse how several machine learning techniques behave in the largest liver transplant database, up to the knowledge of the authors, the database provided by the United Network for Organ Sharing (UNOS) [5]. The opportunity to work with databases including thousands of donor-recipient pairs is crucial, aiming to establish the worldwide applicability of machine learning techniques in the results of LT. In order to have an idea of the magnitude of the database, since 1988, when the database was created, more than 170, 000 liver transplants have been performed representing almost a 22% of all the transplants made in USA, based on Organ Procurement and Transplantation Network (OPTN) data as of January, 2021. This database has been previously considered for some studies [6–10]. However, despite machine learning techniques have demonstrated to be able to obtain good results in several medical areas, some drawbacks are also found when applied to large databases [11–14].

This problem can be tackled from two different points of view: as a classification problem (by discretising the survival time using a set of end-points) or as a Survival Analysis (SA) problem (in which the outcome is the time until the occurrence of an event of interest, in this case, liver graft failure). In this sense, given the huge interest in the SA field, its use was proposed in a previous study of the authors [8]. In this work, we approached the application of SA techniques to the UNOS database. The results achieved in [8] denoted that SA techniques were only able to achieve a low performance in this dataset, showing that the problem is complex. Thus, in the current study, we decided to tackle this problem from a completely different point of view, in this case, as binary classification problems with different end-points.

The main interest of this paper is the application of state-of-the-art machine learning techniques to the largest database of liver transplant, the UNOS database. The importance of this study lies in the interest of obtaining efficient and accurate approaches, applicable in most situations. In this sense, the UNOS collects information not only from donors and recipients, but also from the pre- and post-transplant from all the health care system centres of the USA.

## Materials and methods

All the information and procedures conducted in this studio were in accordance to the ethical standards of both the local Human Research Ethics Committee and the Declaration of Helsinki 1975. The data was originally requested to the UNOS/OPTN website (https://optn.transplant.hrsa.gov/data/request-data/) on 14th October 2015, and a STARFILE dataset (Standard Transplant Analysis and Research) was received, with de-identified patient-level information for transplant recipients and waiting list candidates. Therefore, the data used have been supplied by the United Network for Organ Sharing (UNOS) as the contractor for the Organ Procurement and Transplantation Network (OPTN). Note that patient data was obtained in a fully anonymised and de-identified manner, and that none of the authors of this paper had access to identifying patient information.

### UNOS database: Patient selection and exclusion criteria

For this study we have considered the liver transplantation dataset from the United Network for Organ Sharing (UNOS) database [5]. Although it was founded in March 1984, we have considered those transplants performed from November 2004 onwards, date when the last change of variable formats was made. Partial and split liver transplants, living donor liver transplants and combined liver transplants were excluded from the study. All these transplants, which represent less than 5% of the total, are considered as particular cases, and the donor-recipient matching is usually done following specific criteria. All the recipients older than 18 years were included, and all the patients were followed from the date of transplant until graft-loss prior to five years after transplantation.

In order to make a complete analysis of the liver transplant, we have considered four different end-points (period of time to control graft-loss): three months (3M), one year (1Y), two years (2Y) and five years (5Y). The choice of these end-points has been made by experts [15–17]. Note that graft survival has been defined as the period from transplantation to the time a re-transplant is required or to the time the recipient dies. Deaths not related to the transplantation procedure have been excluded. The initial number of transplants was 39, 189, which decreases for every end-point considered, because some of the transplants are censored, i.e. there is no available information about the outcome for that specific end-point. Table 1 shows the total number of transplants performed for each end-point, as well as their class distribution. An important difference between the different end-points is that the degree of imbalance of the dataset is lower as we consider later end-points, given that the number of non-survival cases increases. For example, in the case of 5Y, 20, 456 transplants are considered, from which 8, 886 belong to the non-survival class, and 11, 570, to the graft-survival class. A notable imbalance degree is shown for all datasets which can lead to trivial classifiers (i.e. classifiers predicting survival for all D-R pairs).

**Table 1:** Number of transplants performed for each end-point and their class distribution.

End-points	Non-Survival class	Survival class	Total transplants
3 months (3M)	2, 928	34, 718	37, 646
1 year (1Y)	5, 180	28, 470	33, 650
2 years (2Y)	6, 614	23, 202	29, 816
5 years (5Y)	8, 886	11, 570	20, 456

### Variable selection

Although the UNOS database includes more than 350 variables, a huge number of them are redundant (most likely due to format changes or important differences in the acquisition of the values), other ones are trivial (i.e. indexes and dates, among others), and a vast quantity of them has a high percentage of missing values (we have kept only those variables with a percentage of missing values lower than 10%). Moreover, other variables were discarded, such as patient address or other information not interesting enough for the medical decision making. As previously stated, the collection of data for large datasets (where different procedures or diverse protocols for obtaining scores or corporal indices are joined in a common database) causes controversy. This could happen not only due to the different mechanisms considered in every health care unit, but also because of the distinct ways to categorise the same patient, causing incongruities. Taking all of this into account, a final set of 28 variables was considered, which is shown in Table 2.

**Table 2:** Main characteristics of the features considered: Name, type and values.

Recipient		
Name	Type	Value
Age (A-R)	Numeric	[18, 83]
BMI (B-R)	Numeric	[15.04, 72.86]
Cardiopathy (CA-R)	Binary	0: absense; 1: presence
CMV (C-R)	Binary	0: absense; 1: presence
Days in Wait list (DW-R)	Numeric	[0, 3516]
Days in ICU (DA-R)	Numeric	[0, 20]
Diagnosis at listing (DI-R)	Nominal	0: alcohol; 1: VHC; 2: VHB; 3: cholestasics; 4: FHF; 5: Others
Diagnosis of the liver (DL-R)	Nominal	0: alcohol; 1: VHC; 2: VHB; 3: cholestasics; 4: FHF; 5: Others
Ethnicity (E-R)	Nominal	0: white; 1: black; 2: hispanic; 3: others
Medical Condition (MC-R)	Nominal	0: in ICU; 1: not hospitalised
MELD (ME-R)	Numeric	[6, 75]
Previous Malignancy (PM-R)	Binary	0: absense; 1: presence
Previous Upper abdominal surgery (PU-R)	Binary	0: absense; 1: presence
Risk factors Portal Vein (R-R)	Binary	0: absense; 1: presence
TIPSS (T-R)	Binary	0: absense; 1: presence
Donor		
Name	Type	Value
Age (A-D)	Numeric	[2, 92]
BMI (B-D)	Numeric	[10.76, 72.42]
Cause of death (COD-D)	Nominal	0: anoxia; 1: cerebrovascular/stroke; 2: head trauma; 3: CNS tumour
CDC risk HIV (CDC-D)	Binary	0: absense; 1: presence
Creat (CR-D)	Numeric	[0.07, 25]
CMV (C-D)	Binary	0: absense; 1: presence
Ethnicity (E-D)	Nominal	0: white; 1: black; 2: hispanic; 3: others
Hepatitis C (HE-D)	Binary	0: absense; 1: presence
History of Hypertension (HH-D)	Binary	0: absense; 1: presence
History of Drugs (HD-D)	Binary	0: absense; 1: presence
Non-heart beating (N-D)	Binary	0: absense; 1: presence
Matching variables at transplant		
Name	Type	Value
ABO compatibility (A-T)	Binary	0: non-compatible; 1: compatible
Gender compatibility (G-T)	Nominal	0: Same gender; 1: Donor male and Recipient female; 2: Donor female and Recipient male

Unknown variables at transplant such as cold ischemia time were not considered, because they should be estimated without any option to establish a real comparison. Moreover, the inclusion of the cold ischemia time would be a handicap, specially for those countries with a huge territorial expansion such as the USA, in which the estimation of the cold ischemia time would be highly inaccurate. In addition, for this kind of post-transplant variables, the prioritisation system in the USA is extremely complex to allow their estimation.

To impute the missing values, the average value in the training set is considered for continuous and quantitative variables, whereas the mode is considered for binary and qualitative variables.

### Experimental settings

To tackle the D-R allocation problem, we have considered different survival prediction methods, which can be divided into two groups: 1) classical statistical methods, such as Logistic Regression (LR) and Naïve Bayes (NB), and 2) standard machine learning techniques, such as Multilayer Perceptron (MLP), Random Forest (RF), Support Vector Machines (SVM), Decision Trees (C4.5), k-nearest neighbours (kNN) and Gradient Boosting (GB). These techniques cover a vast range of methods from the state-of-the-art, including the best performance classifiers. All these methods have been run using scikit-learn framework [18]. Moreover, we compare their results against the standard scores used in the literature (MELD, DMELD, BAR, DRI, SOFT and PSOFT) [19].

Regarding the evaluation process, the Confusion Matrix (CM), the Accuracy (Acc), the Minimum Sensitivity (MS)—i.e. the minimum of the sensitivities for each class–, and the Area Under ROC curve (AUC) have been used. The last three metrics vary between 0 and 1, where, the higher value, the better performance obtained.

To evaluate the results, a 10-fold cross-validation technique has been used. The results are then computed using the mean and standard deviation from the 10 models obtained, and the confusion matrix is computed as the sum of the test confusion matrices obtained for each model.

**Fig 1:**
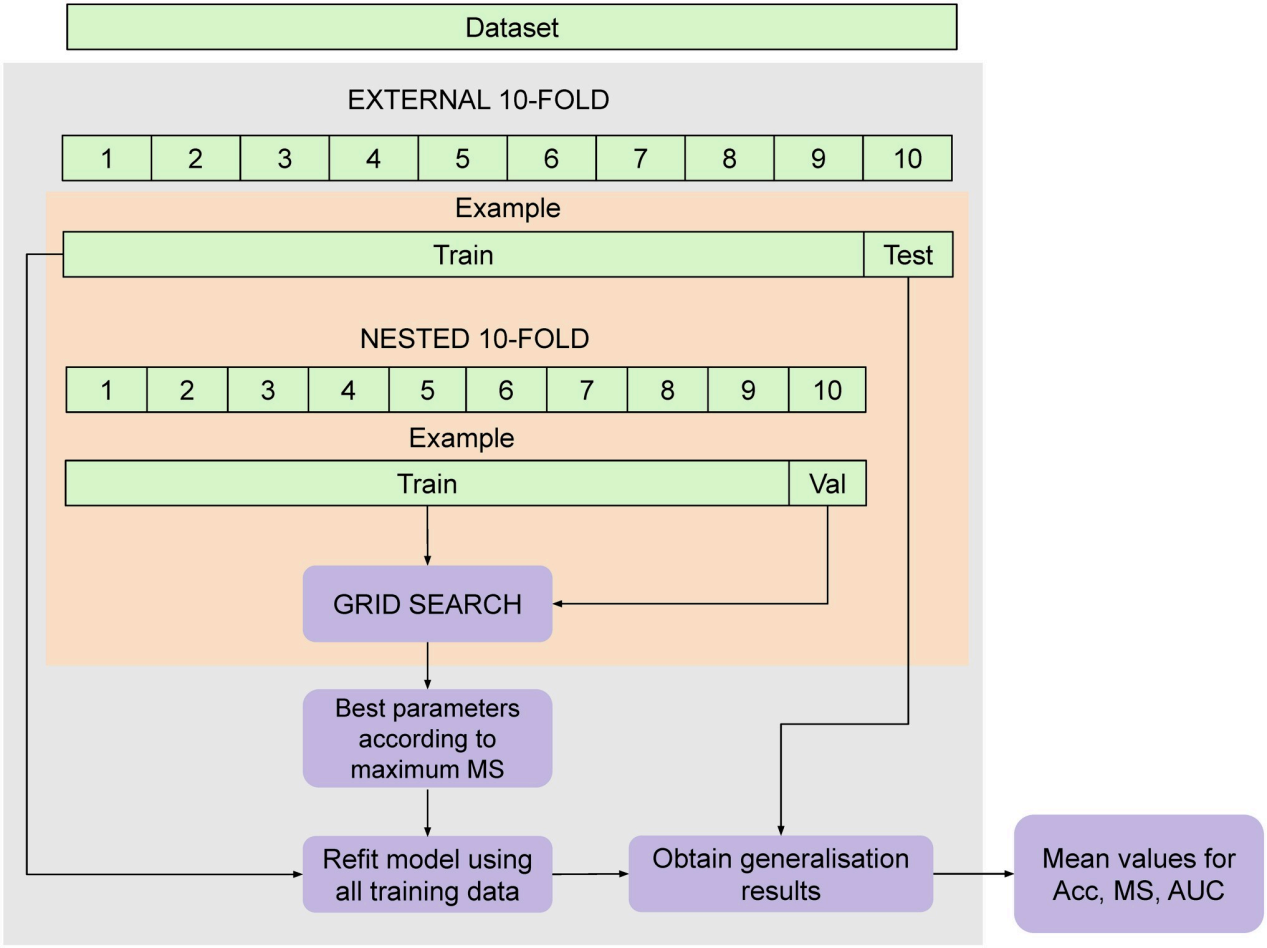
Experimental designed followed.

Furthermore, to establish a robust comparison of the methods, the parameters of each technique have been chosen using another independent 10-fold cross-validation over the training set. The best parameter configuration is the one that obtained the maximum MS in the nested 10-fold cross-validation, because one of the main objectives is to increase the classification rate of the minority class (in this case, the non-survival). Note that the test sets are not used for model selection. The range of parameters used during model selection are specified in Table 3. On the other hand, the whole procedure is clarified in Fig 1.

**Table 3:** Parameters used during the model selection. NB does not involve parameters to be adjusted.

Logistic Regression (LR)	
Regularisation	L1
Inverse of regularization strength	*C* ∈ {10^−6^, 10^−5^, …, 10^2^}
Multilayer Perceptron (MLP)	
L2 penalty (regularization term) parameter	*α* ∈ {10^−7^, 10^−6^, …, 10^−2^}
Number of neurons (1 hidden layer)	*n* ∈ {5, 10, 20, 30, 40, 50}
Maximum number of iterations	*iter* ∈ {500, 1000, 1500}
Random Forest (RF)	
Number of trees	*t* ∈ {10, 100, 1000}
Quality criterion	*Gini* or *Entropy*
Maximum depth for the trees	*d* ∈ {3, 4, 5, 6, 10}
Minimum number of samples per node	*m* ∈ {2, 3, …, 9}
Support Vector Machines (SVM)	
Penalty of the error	*C* ∈ {10^−4^, 10^−3^, …, 10^2^}
Kernel coefficient for *rbf*	*kernel* ∈ {10^−4^, 10^−3^, …, 10^2^}
Decision Trees (C4.5)	
Maximum depth for the trees	*d* ∈ {1, 3, …, 19}
Minimum number of samples per node	*m* ∈ {10, 30, …, 490}
k-Nearest Neighbours (kNN)	
Number of neighbours	*k* ∈ {2, 3, 4, 5}
Gradient Boosting (GB)	
Number of trees	*t* ∈ {10, 100, 1000}
Learning rate	*lr* ∈ {0.1, 0.5, 0.7, 1}
Maximum depth for the trees	*d* ∈ {3, 4, 5, 6, 10}
Minimum number of samples per node	*m* ∈ {2, 3, …, 9}

### Rule-based system

Once the best model is obtained, the D-R matching can be done following a rule-based system, trying to achieve a balance between graft survival and MELD. The procedure of the proposed system is the following: the model allocates a D-R pair, only if the probability of graft-survival is significantly higher than the rest of possible matchings. A probability of graft-survival is considered significantly higher than any other, if the difference between them exceeds the standard deviation of all the probabilities obtained by the model. In the case the model is not able to find significant differences between the possible matches, the recipient with higher MELD is chosen. Moreover, if there are two or more recipients with the same MELD, i.e. a second tie, the donor is allocated to the recipient with the highest number of days in the waiting list.

## Experimental results and validation

### Results

The results obtained are shown in Table 4, where the mean and standard deviation (SD) of the results of the external 10-fold for every classifier and dataset considered (depending on the split-point) are shown.

**Table 4:** Mean±SD of the Acc, MS and AUC evaluation metrics (values between 0 and 1). The time (measured in seconds) is the total time needed for completing the external 10-fold.

3 months end-point				
	Acc	MS	AUC	Time
LR	0.643 ± 0.005	**0.541 ± 0.034**	**0.633 ± 0.017**	4953.802
MLP	**0.905 ± 0.003**	0.033 ± 0.008	0.535 ± 0.014	617814.529
RF	0.685 ± 0.022	0.466 ± 0.030	*0.618* ± *0.018*	4487.515
SVM	0.674 ± 0.009	0.463 ± 0.019	0.609 ± 0.015	823599.329
C4.5	0.585 ± 0.022	*0.520* ± *0.038*	0.577 ± 0.020	*2885.097*
NB	0.821 ± 0.008	0.212 ± 0.016	0.608 ± 0.016	**177.605**
kNN	0.812 ± 0.007	0.145 ± 0.023	0.506 ± 0.012	51480.251
GB	*0.873* ± *0.007*	0.098 ± 0.017	0.519 ± 0.016	68531.756
1 year end-point				
	Acc	MS	AUC	Time
LR	0.618 ± 0.008	**0.555 ± 0.023**	**0.631 ± 0.016**	2782.414
MLP	**0.820 ± 0.004**	0.082 ± 0.015	0.557 ± 0.013	560470.391
RF	0.617 ± 0.020	0.523 ± 0.040	*0.614* ± *0.017*	4028.935
SVM	0.619 ± 0.011	0.531 ± 0.031	0.613 ± 0.015	815035.545
C4.5	0.583 ± 0.022	*0.532* ± *0.025*	0.589 ± 0.009	*1938.553*
NB	0.758 ± *0.005*	0.250 ± 0.009	0.600 ± 0.011	**156.372**
kNN	0.680 ± 0.008	0.278 ± 0.021	0.516 ± 0.011	43251.562
GB	*0.788* ± *0.008*	0.138 ± 0.019	0.542 ± 0.016	59644.420
2 years end-point				
	Acc	MS	AUC	Time
LR	0.610 ± 0.011	*0.558* ± *0.018*	**0.629 ± 0.012**	7445.818
MLP	**0.744 ± 0.006**	0.145 ± 0.010	0.564 ± 0.014	506900.834
RF	0.613 ± 0.011	0.516 ± 0.025	*0.611* ± *0.011*	4363.486
SVM	0.586 ± 0.014	**0.561 ± 0.014**	0.609 ± 0.013	1474150.054
C4.5	0.581 ± 0.018	0.535 ± 0.022	0.585 ± 0.015	*1688.437*
NB	0.695 ± 0.012	0.315 ± 0.020	0.599 ± 0.017	**239.667**
kNN	0.599 ± 0.007	0.384 ± 0.015	0.523 ± 0.008	29809.384
GB	*0.713* ± *0.005*	0.207 ± 0.012	0.561 ± 0.011	51090.888
5 years end-point				
	Acc	MS	AUC	Time
LR	**0.614 ± 0.012**	**0.584 ± 0.017**	**0.654 ± 0.011**	*1695.937*
MLP	0.591 ± 0.013	0.462 ± 0.013	0.599 ± 0.011	161224.104
RF	**0.614 ± 0.014**	0.535 ± 0.013	*0.644* ± *0.019*	2641.192
SVM	0.593 ± 0.017	0.550 ± 0.013	0.624 ± 0.021	566395.494
C4.5	0.582 ± 0.006	*0.553* ± *0.036*	0.609 ± 0.013	*2425.858*
NB	*0.602* ± *0.010*	0.500 ± 0.019	0.622 ± 0.012	**169.528**
kNN	0.539 ± 0.006	0.532 ± 0.005	0.553 ± 0.008	18290.801
GB	0.586 ± 0.009	0.492 ± 0.017	0.600 ± 0.009	30191.146

The best result is highlighted in **bold**; the second one is shown in *italics*.

As can be seen in Table 4, all the methods obtained similar results for all the datasets, but LR stands out, being the one with the performance in terms of MS and AUC, which are the metrics that we give more attention, given that Acc is not a good measure for imbalanced datasets [20]. Especially, focusing on the 5-years-end-point dataset, it can be seen that LR achieved the best performance for all metrics, using the second lowest time. Finally, according to the standard deviations, the models seem to be stable throughout the cross-validation stage.

From the results shown in Table 4, it can be concluded that complex machine learning techniques such as MLP, RF, SVM, C4.5 or GB obtained lightly worse results than LR, which is a standard statistical method. Focusing on the 5-years-end-point, most AUCs are over 0.600, being 0.654 in average for LR, the best result achieved. As we want to measure the longest graft-survival time, from this moment, we are going to focus on the 5-years end-point dataset.

**Fig 2:**
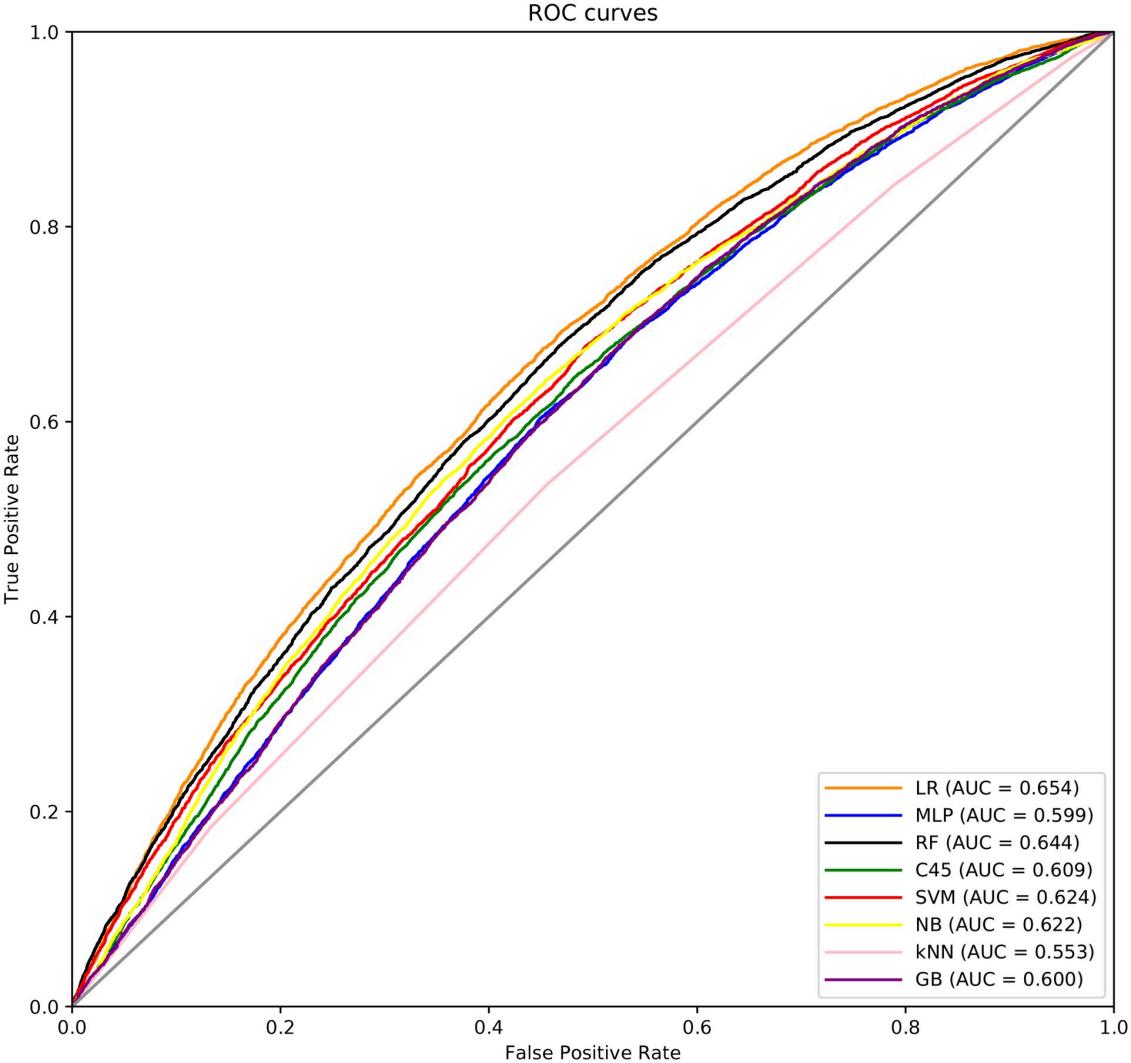
ROC curves for the best models obtained for the 5-years-end-point dataset.

In Fig 2, the ROC curves for the best models of the 5-years-end-point dataset are shown, considering the complete dataset (i.e. the union of the test predictions for the 10 folds). As can be seen, all models performs similarly, and we can differentiate three levels of performance: the best model is the LR, then, RF, SVM and NB perform similarly, followed by, C4.5, GB and MLP that perform equally, and finally, kNN obtained the worse value.

**Fig 3:**
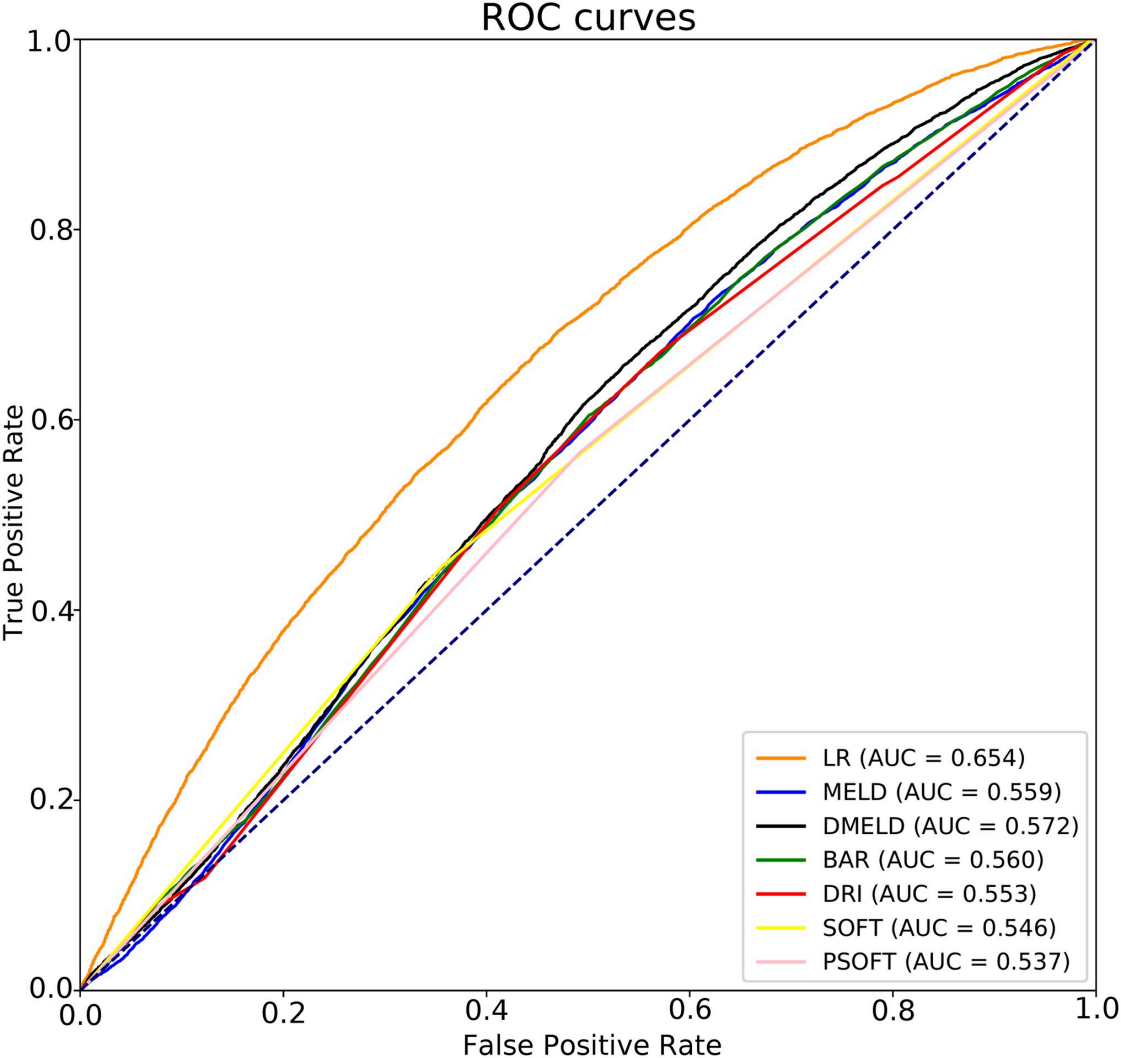
Comparisons of ROC curves for LR vs MELD, D-MELD, BAR, DRI, SOFT and P-SOFT scores on the 5-years-end-point dataset.

A comparison against the state-of-the-art scores MELD, DMELD, BAR, DRI, SOFT and PSOFT [19] is included in Fig 3 to show their predictive capability. It can be seen that there is a big difference between the AUC obtained by the best LR model and the one obtained by other scores, which are close to 0.5 (performance of a random classifier). Note that there is an increase higher than 14% between the best AUC obtained by the state-of-the-art scores (DMELD, 0.572) and the LR model (0.654).

### Interpretation of the best model obtained

In this section, we analyse the best model obtained by the LR, specifically the importance of each variable for the prediction of graft-survival after transplantation (5-year end-point dataset) is studied in detail. The linear weights obtained are included in Table 5. In this table there are 49 variables, due to the decomposition of ordinal and nominal variables (see Table 2) into binary ones. We have considered the absolute value of the weights to rank the variables, and, depending on the sign, we have concluded a positive or negative influence on the graft-survival after transplantation (note that the positive class is the survival at 5 years).

**Table 5:** Best LR model weights per variable (graft-survival prediction at 5-years). The variables are ranked according to the coefficient absolute value.

Rank	Variable	Weight	Rank	Variable	Weight
1	DW-R	−1.916	26	E-D(0)	0.247
2	ME-R	−1.450	27	PU-R	−0.240
3	DA-R	−1.176	28	E-R(2)	0.219
4	A-D	−1.132	29	G-T(1)	0.215
5	A-R	−0.703	30	COD-D(0)	0.203
6	N-D	−0.646	31	DL-R(1)	0.197
7	MC-R	−0.531	32	DI-R(0)	0.184
8	DL-R(2)	0.524	33	E-R(3)	0.179
9	B-R	0.509	34	G-T(2)	0.164
10	R-R	−0.500	35	HD-D	−0.160
11	DI-R(2)	0.493	36	CDC-D	−0.150
12	DI-R(3)	0.491	37	E-D(3)	−0.132
13	PM-R	−0.445	38	B-D	−0.127
14	DI-R(4)	0.445	39	E-D(1)	0.112
15	COD-D(3)	0.411	40	E-R(0)	0.111
16	DL-R(0)	0.404	41	HE-D	−0.109
17	DI-R(5)	0.393	42	T-R	−0.088
18	DL-R(4)	0.383	43	HH-D	−0.082
19	E-R(1)	−0.368	44	CA-R	−0.063
20	COD-D(2)	0.336	45	C-R	0.057
21	DL-R(3)	0.328	46	E-D(2)	0.048
22	A-T	0.303	47	DI-R(1)	0.041
23	G-T(0)	0.286	48	DL-R(5)	0.020
24	COD-D(1)	0.265	49	C-D	0.012
25	CR-D	−0.251			

As we can see in Table 5, the LR model has 49 coefficients. These coefficients are associated to as many characteristics or variables of the donor-recipient pairs, plus a coefficient that is the independent term of the model. The dependent variable is binary with two events: *S* if the graft survives more than 5 years (positive class) or *NS* on the contrary (negative class), following a Bernouilli distribution of parameter *p*.

Although the vast majority of important variables belongs to the recipient, there are some variables of the donor with a great impact on the model, such as the age or whether the donor is non-heart-beating. Those variables that contribute most to the models are the number of days in the wait list (recipient), the MELD (recipient), the number of days in ICU (recipient), the age (both donor and recipient) and whether the donor is non-heart-beating, among others. As can be seen, MELD has a great influence in our model, but the number of days in wait list or in ICU has a similar or greater impact. These findings are consistent with the results in the state-of-the-art, where the age, the number of days in ICU and in wait list are considered important factors for the graft-survival after transplantation.

### Simulation of the rule-based system

In this section, a simulation for the application of the rule-based system described previously is applied. First of all, the LR model chooses one D-R pair over the others, only if the difference between their probabilities of graft-survival is higher than 14%, since the standard deviation of all the probabilities obtained by the LR model is 0.139. For this simulation, 5 recipients and 10 donors are randomly selected, and we consider three scenarios: 1) selecting 5 recipients with a MELD in the range [23, 27] (Table 6), 2) selecting those with MELD [34, 40] (Table 7), 3) instead of applying restrictions to MELD, we consider Extended Criteria Donors (ECDs), that is, donors over 70 years or with a BMI over 40, among others (Table 8). For visualisation purposes, those receptors which do not show significant differences with respect to the maximum graft-survival probability are shadowed. Bold face indicates selected values from all statistically significant. Finally, the chosen recipient identifier can be seen in the last row.

**Table 6:** First simulation of D-R allocation by the best LR model (recipients MELDs 23 − 27).

*R* − *MELD*	*D*1	*D*2	*D*3	*D*4	*D*5	*D*6	*D*7	*D*8	*D*9	*D*10
*R*1 − 27	0.379	**0.540**	0.453	**0.523**	0.325	0.459	0.442	0.359	**0.547**	0.585
*R*2 − 26	0.293	0.444	0.360	0.427	0.247	0.366	0.350	0.276	0.451	0.490
*R*3 − 25	**0.410**	0.572	0.485	0.554	0.354	0.491	**0.474**	**0.389**	0.578	0.616
*R*4 − 24	0.411	0.573	**0.561**	0.555	0.427	**0.568**	0.475	0.390	0.579	**0.686**
*R*5 − 23	0.522	0.677	0.667	0.661	**0.612**	0.673	0.586	0.500	0.683	0.773
Allocation	*R*3	*R*1	*R*4	*R*1	*R*5	*R*4	*R*3	*R*3	*R*1	*R*4

**Table 7:** Second simulation of D-R allocation by the best LR model (recipient MELDs 34 − 40).

*R* − *MELD*	*D*1	*D*2	*D*3	*D*4	*D*5	*D*6	*D*7	*D*8	*D*9	*D*10
*R*1 − 40	**0.492**	0.337	0.411	0.393	0.259	0.308	**0.396**	0.402	0.245	**0.464**
*R*2 − 39	0.525	**0.440**	**0.519**	0.500	0.351	**0.407**	0.428	0.509	0.334	0.497
*R*3 − 38	0.622	0.465	0.543	**0.524**	0.374	0.431	0.527	**0.534**	0.356	0.596
*R*4 − 35	0.610	0.528	0.605	0.658	0.510	0.494	0.515	0.666	0.491	0.584
*R*5 − 35	0.616	0.533	0.611	0.663	**0.516**	0.500	0.521	0.671	**0.497**	0.590
Allocation	*R*1	*R*2	*R*2	*R*3	*R*5	*R*2	*R*1	*R*3	*R*5	*R*1

**Table 8:** Third simulation of D-R allocation by the LR best model (extended criteria donors).

*R* − *MELD*	*D*1	*D*2	*D*3	*D*4	*D*5	*D*6	*D*7	*D*8	*D*9	*D*10
*R*1 − 36	0.497	**0.479**	0.493	**0.493**	0.489	0.566	0.318	**0.608**	0.535	0.538
*R*2 − 28	**0.535**	0.441	0.531	0.455	0.527	**0.603**	0.352	0.571	**0.573**	0.575
*R*3 − 25	0.528	0.510	0.525	0.524	0.520	0.596	0.346	0.637	0.566	0.569
*R*4 − 23	0.553	0.536	**0.550**	0.549	**0.546**	0.621	0.369	0.660	0.591	0.593
*R*5 − 21	0.675	0.588	0.672	0.601	0.668	0.733	**0.571**	0.706	0.708	**0.768**
Allocation	*R*2	*R*1	*R*4	*R*1	*R*4	*R*2	*R*5	*R*1	*R*2	*R*5

Table 6 shows the first simulation. For this case, 5 recipients with MELD 23 − 27 and 10 donors are randomly selected. It can be seen that, when donor *D*1 is offered, following a MELD-driven approach, the recipient *R*1 would be chosen. However, considering the best LR model proposed, the recipient *R*3 would be considered for allocation, because the difference between both probabilities of graft-survival is significant. A similar situation happens for donors *D*3, *D*5 − *D*8 and *D*10. In the case of *D*2, the MELD-driven approach and the LR model lead to the same allocation, since *R*1 belongs to the group of better probabilities of graft-survival and has the highest MELD (this pattern is repeated for *D*4 and *D*9). Note that the rule-based system lays on the basis of MELD-allocation, but, when the probabilities show significant differences, a lower-MELD recipient can be chosen. Therefore, a D-R pair is chosen only in cases of real biological (not mathematical) differences.

Table 7 shows the simulation with recipients with MELD 34 − 40. The same strategy is applied to high-MELD recipients. Focusing on *D*5 and *D*9, it is interesting to remark that, when two probabilities of graft-survival do not show significant differences and there is a tie in MELD, the recipient will be that with the longest time in the waiting list, in this case, *R*5. As in the previous simulation, the decision is only taken by the mathematical model when there are significant differences (*D*2 − *D*6, *D*8 and *D*9). Otherwise, the decision is made following the MELD score (*D*1, *D*7 and *D*10).

Finally, a last simulation including Extended-Criteria Donors (ECD) is shown in Table 8. Transplants with ECD have been performed successfully for a number of years. In this simulation, the first 5 donors are older than 70, whereas the remaining 5 have a BMI higher than 40, representing a 4.38% and a 3.43%, respectively, of all the donors included in this study. The same analysis done for previous simulations could be also applied to this situation.

## Discussion

To our knowledge, this is the first work that addresses Donor-Recipient (D-R) matching in Liver Transplantation (LT) using the UNOS data set. D-R matching has become one of the most challenging topics in LT in the last years. Unfortunately, standard scorers, such as MELD, SOFT or BAR, fail to consider both mortality in waiting list and benefit survival. These two objectives are difficult to meet, since these metrics pose them as conflicting objectives. A decrease in mortality in the waiting list leads in many cases to worse post-transplant survival results; and, vice versa, obtaining better results may affect the opportunity to be transplanted for the sickest one on the waiting list.

D-R matching is considered as a classification problem, and, for this, variables of the donor, variables of the listed recipients and surgical and logistical aspects are considered to assess the best matching possible [4], which can be based on the survival of the graft, the survival of the recipient, or both. A common problem of the available scores is a remarkably basic statistical methodology that only considers isolated variables with single random graft or patient survival end-points. The combination of several variables and end-points in the setting of artificial intelligence-based decisions that avoid human-guided bias may be the basis for D-R matching and grafts allocation in the future. In 2014, we tested Artificial Neural Networks (ANNs) in the complex scenario of D-R matching with D-R pairs from 11 Spanish transplant units [15]. This study demonstrated that ANNs are a valuable tool for organ allocation to obtain the best benefit of survival. In the current scenario of graft scarcity and waiting list deaths, the absence of a definitive and objective system for liver-donor assignment is unacceptable. After that, we validated ANN methodology in D-R matching in a different health care system (data from King’s College Hospital, KCH), showing that it would be a powerful tool for D-R matching in comparison to other current models [21]. This methodology has been recently validated using gradient boosting and random forest classifiers [22] using data from 272 different centres, denoting that outstanding results could be obtained independently of the population location.

The main goal of this paper was to analyse the behaviour of machine learning techniques applied to the largest liver transplant database, provided by the UNOS [5]. Working with large databases is a great opportunity to achieve a worldwide application of machine learning techniques in the results of LT.

Machine learning methods lead in general to excellent results when combined with a huge amount of information. As an example, Electronic Health Records (EHRs) have been developed to speed up the mechanism for clinician decision making, based on information extracted from these records [23]. However, it has been demonstrated that, for large databases, machine learning algorithms are not always capable of reaching notable results, what can be caused for several reasons [13, 24, 25]: 1) missing values and the imputation techniques used, in combination with the need of clear guidelines regarding how to cope with attributes and patterns with different percentages of missing data, 2) the increasing quantity of different categories for some attributes, which makes the classifier lose accuracy, as well as, 3) the increasing number of Non Specified (NS) cases in this attributes, where some specific information is discarded, since no category matches the particular situation, 4) by contrast, attributes with several categories but a small number of cases per category, make null contribution, and finally, 5) the vast amount of subjective attributes manually introduced may cause incongruities between different expert opinions.

The results we have obtained in the present study include much of the problems described previously. Indeed, most AUCs are over 0.600, being 0.654 in average for LR, the best performance model. These results contrast with those obtained in previous models form the Spanish data set and its subsequent validation in the King’s college Hospital dataset. However, in a similar study made with the UNOS dataset in heart transplantation, Miller et al. [13] have found a lack of improvement of advanced analytic algorithms, as we have described in the present study, concluding that prognostic abilities of machine learning techniques may be limited by quality of the clinical dataset. More recent studies [25, 26] have also demonstrated no evidence of performance benefit for machine learning methods over logistic regression.

In the dataset considered in the present study, a vast amount of missing data were found. Not all the regional centres give the same importance to the data collection step for the database. Hence, the curation of the database is a tedious procedure due to the large percentage of missing values. The imputation of data makes the database lose veracity and robustness, leading to worse performances because of data granularity and quality. Furthermore, the entries of the database are collected by 11 regional centres. This process lacks from consistency, caused by diverse reasons: 1) the administrative centres provides their original data to a global database, being possible to give different formats to the variables from the other centres. 2) In addition, a given variable could be obtained following different procedures or index measures. 3) Finally, the database may include incongruities because a given situation could be categorised contradictorily. The inclusion of the cold ischemia time has arisen much controversy in the literature [27, 28]. Although it has been considered an important variable, it is a post-transplant variable, for which a priori information is not known. Estimating the cold ischemia time to predict donor-recipient matching is a challenging task, because this estimation has to be done with no prior information but the cities of origin of both patients (which in the case of USA is arduous, given the large distances between the hospital of different states) and the strategy to allocate organs between all the regional centres.

Finally, it should be discussed the trajectory of different scores considered along the years [29, 30]. The D-R matching has been performed following the guidelines proposed by scores with different goals published in the literature. With the exception of the LR model, the results obtained in the present study did not differ in essence from those obtained with the classic scores (MELD, BAR, SOFT, etc.) based on conventional biostatistics. This does not mean a lack of usefulness of artificial intelligence in the problem of D-R matching in liver transplantation, but the importance of emphasizing the need for well-designed and well-constructed databases, and, of course, filled with diligence and professionalism.

The main goal of machine learning is to provide the medical community with a tool bridging the gap between the medical decision (subjectivity) and strict mathematical scores (objectivity). For this purpose, a rule-based system is proposed for the management of the waiting list for liver transplant. This system is objective (does not include human subjectivity in the selection of the recipient), optimal (it is able to increase the post-transplant survival rates) and, finally, fair, because, if the model does not appreciate a significant difference between two recipients, the organ is allocated to the recipient with the most advanced disease (highest MELD). A deep analysis have been done to increase the most the understanding of the mathematical model and its consistency with the medical findings so far.

## Conclusion

In this paper, a deep analysis of the UNOS database regarding liver transplant is presented. The best results are obtained by the Logistic Regression (LR) method, while machine learning techniques do not live up to their expectation. One of the reasons behind this is the lack of accuracy and robustness needed for machine learning methods to capture essential uncovered features of the problem tackled, although they have obtained outstanding results in other medical-related fields. Despite UNOS is considered a robust clinical registry, it is based on administrative data, making the database suffer from the problem of subjectivity, i.e., given two similar situations, two different choices are made. The extensive analysis made us prove these limitations, since all the methods applied almost had the same predictive ability.

However, as we believe that the techniques have done their best for the D-R matching in liver transplant, an interpretation of the LR best model obtained is done, as well as, several simulations of the organ allocation are included in different scenarios: medium-MELDs, high-MELDs and using extended-criteria donors. To our knowledge, this is the first study comparing different predictive methods in patients undergoing liver transplantation. Future work is required to improve the quality of available data, especially on large databases such as UNOS, in which the amount of information is huge and the curation step must be rigorous and severe. Moreover, reformulating the survival prediction problem into correlated binary classification problems as in [31] will also be considered as future work.
